# The associations of plasma carotenoids and *α*-tocopherol concentrations with fasting glucose in Cameroon

**DOI:** 10.1017/S0007114526106503

**Published:** 2026-06-14

**Authors:** Camille M. Mba, Albert Koulman, Kerry S. Jones, Nita G. Forouhi, Fumiaki Imamura, Sarah R. Meadows, Felix Assah, Jean Claude Mbanya, Nicholas J. Wareham

**Affiliations:** 1 MRC Epidemiology Unit, University of Cambridge, UK; 2 Department of Public Health, Faculty of Medicine and Biomedical Sciences, University of Yaounde I, Yaounde, Cameroon; 3 Nutritional Biomarker Laboratory, MRC Epidemiology Unit, University of Cambridge, Cambridge, UK; 4 Department of internal medicine and specialities, Faculty of Medicine and Biomedical Sciences, University of Yaounde I, Yaounde, Cameroon

**Keywords:** Carotenoids, *α*-tocopherol, Fruits, Vegetables, Glucose

## Abstract

Previous estimations of the associations between fruits and vegetables (F/V) intake with diabetes markers showed mixed results, possibly partly because of the subjective assessment of dietary intake. We aimed to examine the relationship between plasma carotenoids and *α*-tocopherol (as objective markers of F/V intake) and fasting glucose. This was a population-based cross-sectional study in 592 adults in Cameroon. Self-reported F/V intake was assessed using the WHO STEPS questionnaire, and the biomarkers were analysed by HPLC. The mean age of participants was 38·5 (sd 8·6) years (63·7 % women). The median (IQR) number of times participants self-reported consuming fruits in a typical week was 2(1–5) and vegetables was 4(2–7) times/week. Plasma total carotenoids was positively correlated with self-reported intake of fruits (*r* = 0·13) and vegetables (*r* = 0·29), both *P*-values < 0·01. In unadjusted analysis, the difference in fasting glucose comparing the highest against the lowest tertile of the biomarkers concentrations was −0·28 (95 % CI −0·56, −0·001) mmol/l for total carotenoids and −0·31 (–0·59, −0·03) mmol/l for plasma *α*-carotene. The associations became stronger after adjusting for sociodemographics, lifesyle factors and cholesterol (–0·36 (–0·73, −0·002) mmol/l for total carotenoids and −0·41 (–0·79, −0·03) mmol/l for *α*-carotene). There was no evidence of an association between *α*-tocopherol and fasting glucose. We showed an inverse association of total carotenoids and *α*-carotene, objective indicators of F/V intake, with fasting glucose; suggesting that higher F/V intake may be beneficial for diabetes prevention in African populations where F/V intake is low.

The 2017 Global Burden of Disease Study estimated that 11 million deaths globally were attributable to dietary risk factors, with CVD and diabetes among the leading causes of diet-related deaths. Specifically, low intake of fruit and vegetables accounted for 3·9 million global deaths^(1)^. The incidence of type 2 diabetes has risen substantially over the past 3 decades, and identifying modifiable risk factors for public health interventions is a major priority^(2)^.

A higher intake of fruit and vegetables is widely promoted for the prevention of non-communicable diseases including type 2 diabetes^(3)^. However, epidemiological studies on the associations between fruit and vegetable intake and type 2 diabetes are inconclusive, partly owing to the use of dietary assessment methods, which are subjective and prone to measurement error and recall bias^(4–6)^. In a meta-analysis of twenty-three cohort studies, high compared with low intake of fruit and vegetables combined was associated with a 7 % lower risk of type 2 diabetes, but a higher diabetes risk was observed for intakes of some subtypes of fruits and vegetables^(7)^. Low consumption of fruit and vegetables is common worldwide (about 100 g/d of fruits and 200 g/d of vegetables)^(1)^. The Prospective Urban Rural Epidemiology (PURE) study showed that fruits and vegetables are less affordable in low- and middle-income countries than in high-income countries^(8)^. Only few studies so far have assessed the associations of fruits and vegetables intakes using objective measures with type 2 diabetes.

Objectively measured nutritional biomarkers offer a complementary approach to assessing dietary intake since they do not rely on the participant’s memory. Evidence from observational and experimental studies mostly from Western settings suggests that circulating carotenoids and vitamin C, widely found in fruits and vegetables, are valid biomarkers of intake of fruit and vegetables^(9–12)^. *α*-tocopherol, although mostly found in plant seeds and their derivatives such as vegetable oils, is also found in fruits and vegetables such as avocado, mango, pumpkin and spinach. As such, lipid-adjusted *α*-tocopherol has also been suggested as a biomarker of fruit and vegetable intake, although less consistently than circulating vitamin C and carotenoids^(10)^. Observational studies of the association between biomarkers estimating fruit and vegetables intake and type 2 diabetes show conflicting results^(13–16)^. The apparent discrepancy in study findings may suggest differences in the fruit and vegetable sources of the biomarkers, food matrices and food preparation, which vary across populations. Despite the advantages of biomarkers, the use of biomarkers has not been widely applied to test diet–diabetes associations in African settings probably owing to the high cost of analysis of nutritional biomarkers. In fact, we did not find any studies that used nutritional biomarkers to estimate fruit and vegetables intake associations with diabetes markers conducted in Africa, where it is estimated that 79·1 % of adults consume less than the recommended five portions a day of fruits and vegetables^(17)^.

Improving understanding of the association between intake of fruit and vegetables and type 2 diabetes, particularly in populations where there is a high proportion of inadequate intake of fruit and vegetables using objective assessment methods, is important. Low intakes of fruits and vegetables have been reported in Cameroon; in 2003, results from the Cameroon WHO STEPS showed that fruits and vegetables were consumed only about 3 d a week^(18)^. The study presented here aimed to examine the associations of plasma carotenoids and *α*-tocopherol reflecting intake of fruit and vegetables with fasting glucose in rural and urban settings of Cameroon. As a secondary objective of this study, we aimed to assess the correlation of self-reported fruit and vegetable intake with circulating carotenoids and *α*-tocopherol.

## Methods

### Study population and design

This was a population-based cross-sectional study conducted in four sites in rural and urban Cameroon. The urban sites were Yaoundé, the capital region of Cameroon (Centre region), and Bamenda (Northwest region), and the rural sites were Mbankomo (Centre region) and Bafut (Northwest region). The methods used to enrol participants in this study were described elsewhere^(19)^. Briefly, all adults aged 25–55 years without a history of diabetes or CVD were approached through door-to-door recruitment. A total of 651 participants agreed to take part in this study. Results are presented for the 592 participants with blood samples available for analysis (online Supplementary Figure 1). Ethical clearance was sought and obtained from the Cameroon National Ethics Committee, and all participants provided written informed consent before inclusion in this study.

### Data collection

Participants who had provided informed written consent were invited to attend the session at a testing facility established for the study in their local hospital. Data collection at the facility was done by trained field staff. All participants provided blood samples in the morning between 07.30 and 09.30 after an overnight fast of at least 8 h. Fasting glucose and 2-h post 75 g oral glucose tolerance test were measured on fresh capillary whole blood using a HemoCue B-Glucose Analyzer (HemoCue AB) onsite.

Blood samples collected from all the participants were immediately refrigerated at 4°C. The samples were centrifuged at about 1400 *g* at 4°C for 10 min within 24 h of blood collection, and plasma aliquots were stored at −80°C. Plasma samples were transported on dry ice by air to Cambridge, UK, and stored at −80°C until analysis.

#### Measurement of plasma carotenoids and tocopherol

Plasma carotenoids and *α*-tocopherol were analysed at the Nutritional Biomarker Laboratory, MRC Epidemiology Unit, University of Cambridge, by HPLC coupled with a photodiode array detector (HPLC-PDA) using analytical methods described previously^(20)^.

Briefly, plasma samples were deproteinised with methanol followed by liquid-liquid extraction in hexane. The samples were dried and reconstituted in ethanol and acetonitrile in two steps. The samples were injected onto the HPLC (Waters Acquity, Waters UK), and chromatographic separation was performed with a YMC-Pack Pro C18 analytical column: 3 µm, 4·6 mm × 150 mm (YMC Europe Ltd) and with a 25:75 ethanol + 0·1 % trimethylamine: acetonitrile + 0·1 % triethylamine mobile phase at 1·2 ml/min. Detection was accomplished by a photodiode array detector. Plasma concentrations of six carotenoids (*α*- and *β*-carotene, lycopene, *β*-cryptoxanthin, lutein and zeaxanthin) and *α*-tocopherol were determined. Lutein and zeaxanthin were not differentiated. Carotenoids were quantified at 450 nm and tocopherol at 300 nm. Internal standards (tocopherol acetate and apo-8’-carotenal) were added prior to the extraction step to normalise the sample preparation process and for instrument detection variability. The ratio of the signal of the metabolite to the internal standard obtained was compared against that of a calibration curve to determine the concentration of the individual biomarkers. Samples preparation was conducted in a darkened room under non-actinic lighting to prevent degradation of *α*-tocopherol and carotenoids due to their photosensitive nature. The intra % CV for tocopherol acetate was 4·3–9·8 % and for apo-8-carotenal was 3·9–9·5 %. The recovery of each internal standard from pervious calculations is 74–84 % for tocopherol acetate and 76–96 % for apo-8-carotenal.

We imputed random values between zero and the lower limit of quantification for biomarkers with < 5 % of missing data (0·17 % *α*-tocopherol, 2·87 % lutein + zeaxanthin and 4·9 % for lycopene). *β*-cryptoxanthin was not included in the analysis because of the high proportion of values below the limit of quantification (> 30 %).

#### Measurement of lipid markers

Cholesterol and TAG were measured at the National Institute for Health Research (NIHR) Cambridge Biomedical Research Centre (BRC), Core Biochemical Assay Laboratory. Total cholesterol, HDL-cholesterol and TAG were measured by the enzymatic method using automated assays on the Dade Behring Dimension RxL analyser. LDL concentrations were derived by the Friedewald formula (LDL = total cholesterol – (TG/2·2) – HDL), when TAG levels were < 4·5 mmol/l.

#### Assessment of fruit and vegetable intake

Using an adapted version of the WHO STEPwise approach to Surveillance (STEPS) questionnaire^(21)^, trained interviewers collected data on intake of fruit and vegetable. Participants were asked four questions relating to the frequency of fruit and vegetable intake: (i) the number of days in a typical week when they eat fruit or (ii) vegetables and (iii) the number of times in a typical week they eat fruits or (iv) vegetables.

#### Covariate measurement

Data on sociodemographics (age, sex, education level, rural or urban residence) and health behaviours (alcohol intake, smoking and physical activity) were also collected by interviewers using an adapted version of the WHO STEPS questionnaire for fruit and vegetables intake^(21)^. Based on responses to the questions ‘Have you ever smoked any tobacco product/consumed a drink that contains alcohol?’ and ‘Do you currently smoke any tobacco product/did you consume a drink that contains alcohol within the past 12 months?’, smoking status and alcohol intake were categorised as never, past or current.

Waist circumference was measured to the nearest 0·1 cm in participants wearing light clothing using a non-stretch fibreglass tape at the level of the midpoint between the lower costal margin and the anterior superior iliac crests and height measured using a standard rigid stadiometer. Body weight and composition were measured using electronic scales and bioelectrical impedance (Tanita TBF-531 scales; Tanita UK), respectively. BMI (in kg/m^2^) was computed as the body weight (kg) divided by the square of height (m^2^).

Physical activity data were collected with self-reported and objective methods in all participants. Self-reported activities at work, recreational activities and travel were recorded using the Global Physical Activity Questionnaire (GPAQ) estimates of energy expenditure in each domain in metabolic equivalents of task (MET)-min/week and physical activity energy expenditure (GPAQ PAEE) derived^(21)^. Objectively measured PAEE was measured using a combined heart rate and movement sensor (Actiheart; Cambridge Neurotechnology) over seven continuous days which had been previously validated against the criterion of doubly labelled water in this population^(22)^. Heart rate responses during a step test were used for the individual calibration of the heart rate data. PAEE was scaled for body weight and expressed as KJ/kg/d. Using the time spent in minutes per day at different intensities of physical activity, we created three categories: < 1·5 MET, sedentary behaviour; 1·5–3 MET, light physical activity; > 3 MET, moderate-to-vigorous physical activity.

### Statistical analysis

All statistical analyses were performed using STATA 15 (Statacorp). Using the date of the blood draw, we derived the season of measurement of the biomarkers as long dry (December–March), light rain (April–May), short dry (June–July) and heavy rain (August–November). We calculated plasma total carotenoids as the sum of plasma *α*- and *β*-carotene, lycopene, lutein and zeaxanthin. The Spearman correlation coefficient was calculated between plasma concentrations of total and individual carotenoids, *α*-tocopherol and self-reported intake of fruits and vegetables. We fitted multiple linear regression models adjusted for age and sex to identify the potential correlates of continuously distributed plasma carotenoids and *α*-tocopherol after log-transforming the values of plasma total carotenoids, *β*-carotene and lycopene to account for their skewed distributions.

Plasma total and individual carotenoids and *α*-tocopherol were the exposures of interest, and fasting glucose was the outcome of interest. We fitted restricted cubic splines with three knots corresponding to the 25th, 50th and 75th percentiles of continuously distributed biomarkers using model 3 to model any non-linear association between the biomarkers and fasting glucose. We further categorised plasma carotenoids and *α*-tocopherol into tertiles and fitted linear regression models to assess their associations with fasting glucose. Using a block-wise approach, three models, incrementally adjusted for potential confounding variables based on biological plausibility, were fitted. After fitting crude regression models, we further adjusted for age (continuous) and sex (model 1), and then for smoking (never, past or current), alcohol intake (never, past or current), level of education (less than primary school, completed primary school, secondary school and university), residential site (four sites), PAEE (continuous) and BMI (model 2) and total cholesterol (model 3). *P*-values for trend were obtained from linear regression models including the biomarkers as an ordinal variable across tertile categories. With missing information observed for some covariates (PAEE, *n* 50; total cholesterol, *n* 26), complete-case analyses were performed.

We tested for effect modification by sex, rural–urban area of residence, BMI categories and smoking status on the association of plasma carotenoids and *α*-tocopherol with fasting glucose and performed subgroup analysis if the *P*-value for the interaction term was < 0·05. In sensitivity analyses, we imputed missing values of covariates using multiple imputation by chained equations to assess the impact of the missing data on the associations of plasma carotenoids and *α*-tocopherol with fasting glucose. We used multiple imputation by chained equations to create ten multiply imputed datasets and used Rubin’s rule to combine estimates across the imputed datasets^(23)^.

## Results

Of the 592 participants (mean age 38·5 (sd 8·6) years) enrolled in this study, 63·7 % were women and 54·1 % were from urban areas. The descriptive characteristics of participants, stratified by sex and residential site, are presented in online Supplementary Table 1. BMI, systolic and diastolic blood pressure and HDL-cholesterol were higher in urban compared with rural residents. These characteristics have been discussed in depth previously^(24)^.

**Figure 1.**
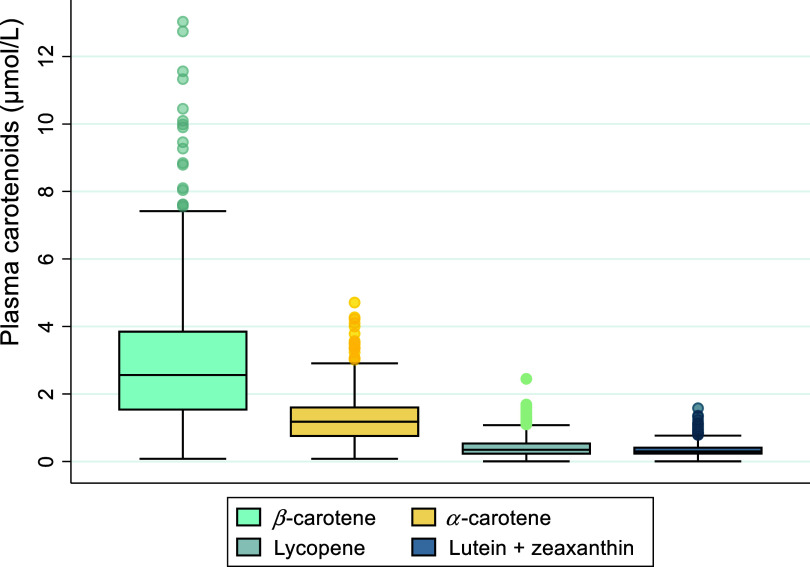
Distribution of plasma individual carotenoids concentrations (µmol/l), Cameroon study, *n* 592.

The median (IQR) of plasma total carotenoids was 4·5 (2·9–6·4) µmol/l, and the mean (sd) of *α*-tocopherol was 19·7 (sd 5·6) µmol/l. Participants living in urban areas had higher plasma concentrations of total carotenoids (4·9 (3·1–6·8) µmol/l) and *α*-tocopherol (20·3 (sd 5·9) µmol/l) than those living in rural areas (total carotenoids: 4·2 (2·5–6·1) µmol/l, *α*-tocopherol: 19·0 (sd 5·2) µmol/l), with a *P*-value < 0·01 for both comparisons. Women had higher plasma total carotenoids (4·9 (3·2–6·9) µmol/l) and *α*-tocopherol (20·4 (sd 5·9) µmol/l) than men (total carotenoids: 3·9 (2·3–6·0) µmol/l, *α*-tocopherol: 18·6 (sd 5·0) µmol/l), with a *P*-value < 0·001 for both comparisons. The distribution of the individual plasma carotenoids is shown in Figure 1, which includes data from the UK National Diet and Nutrition Survey (NDNS) for comparison. The NDNS is a cross-sectional population-based survey providing data on dietary intakes and nutritional status biomarkers of children and adults in the UK, on a nationally representative sample. The NDNS data (collected between 2008 and 2012; years 1–4 of the survey) are presented to enable comparison with a European population. We used identical analytical methods as in the NDNS, and thus comparison between datasets is not affected by between-method differences.

The median (IQR) number of times participants self-reported consuming fruits in a typical week was 2 (1–5) times/week, and the comparable figure for vegetable intake was 4 (2–7) times/week. Participants living in rural areas, and women, reported a higher frequency of fruit consumption (3 (1–6) times/week) than urban residents and men (2 (1–4) times/week), with a *P*-value for both comparisons < 0·01. Similarly, the frequency of self-reported vegetable intake was higher in rural residents (5 (2–9) times/week) and women (4 (2–8) times/week) than urban residents (3·5 (2–6) times/week) and men (3 (2–6) times/week), with a *P*-value < 0·001 for both comparisons.

Plasma total carotenoids, individual carotenoids and *α*-tocopherol were positively correlated with each other (Table 1). Plasma *β*-carotene was the highest contributor to total carotenoids (58·9 %) followed by *α*-carotene (25·5 %) (online Supplementary Figure 2). Plasma total carotenoids and *α*- and *β*-carotene were positively correlated with self-reported intake of fruit with correlation coefficients (*r*) of 0·13, 0·14 and 0·17, respectively (*P*-value all < 0·01). In contrast, plasma lycopene was negatively correlated with self-reported intake of fruit (*r* = –0·12, *P*-value < 0·01) (Table 1).

**Table 1.** Pairwise correlation of individual biomarkers and fruit and vegetable intake (Cameroon study, *n* 592) Table 1 long description.

	*α*-carotene	*β*-carotene	Lycopene	Lutein + zeaxanthin	*α*-tocopherol	Fruit intake	Vegetable intake
Total carotenoids	0·97^‡^	0·98^‡^	0·46^‡^	0·62^‡^	0·47^‡^	0·13^†^	0·29^‡^
*α*-carotene		0·95^‡^	0·38^‡^	0·56^‡^	0·41^‡^	0·14^†^	0·32^‡^
*β*-carotene			0·32^‡^	0·53^‡^	0·39^‡^	0·17^‡^	0·35^‡^
lycopene				0·42^‡^	0·57^‡^	–0·12*	–0·29^‡^
Lutein-zeaxanthin					0·47^‡^	0·06	0·13^†^
*α*-tocopherol						–0·04	–0·03
Fruit							0·32^‡^

Results are Spearman’s correlation coefficients.

*P-value < 0·05.

^†^ *P*-value < 0·01.

^‡^ *P*-value < 0·001.

Plasma total carotenoids and *α*- and *β*-carotene showed stronger positive correlations with self-reported intake of vegetables with correlation coefficients (*r*) of 0·29, 0·32 and 0·35, respectively (*P*-value all < 0·001). Plasma lutein-zeaxanthin was also positively correlated with self-reported vegetable intake (*r* = 0·13, *P*-value < 0·01), whereas lycopene showed a negative correlation with self-reported intake of vegetables (*r* = −0·29, *P*-value < 0·01) (Table 1).

After adjusting for age and sex, factors that were positively associated with plasma total carotenoids and *α*-tocopherol were age (adjusted for sex only), living in urban areas, level of education, the season of light rain and self-reported vegetable intake (for total carotenoids only), self-reported physical activity and plasma cholesterol concentrations (Table 2). The male sex and season of heavy rain were inversely associated with both plasma total carotenoids and *α*-tocopherol. Current smoking status was inversely associated with plasma total carotenoids, while self-reported intake of fruit and vegetables was inversely associated with plasma *α*-tocopherol. Factors positively associated with self-reported intake of fruit and vegetables were the season of light rain and physical activity, and factors associated inversely were the male sex (adjusted for age only), living in urban areas and BMI (online Supplementary Table 2).

**Table 2.** Factors affecting plasma carotenoids and *α*-tocopherol (Cameroon study, *n* 592) Table 2 long description.

	Total carotenoids		*α*-carotene		*β*-carotene		Lycopene		Lutein + zeaxanthin		*α*-tocopherol	
Biomarkers (µmol/l)	*β*-coefficient	95 % CI	*β*-coefficient	95 % CI	*β*-coefficient	95 % CI	*β*-coefficient	95 % CI	*β*-coefficient	95 % CI	*β*-coefficient	95 % CI
Age (10 years)	0·60	0·30, 0·90	0·10	0·05, 0·20	0·20	0·09, 0·30	0·07	–0·009, 0·20	0·05	0·03, 0·07	1·8	1·3, 2·3
Men	–0·89	–1·37, –0·40	–0·16	–0·28, –0·04	–0·40	–0·55, –0·24	0·11	–0·03, 0·25	–0·02	–0·06, 0·01	–1·29	–2·16, –0·44
Urban (*v*. rural)	0·75	0·28, 1·23	0·22	0·10, 0·33	0·49	0·12, 0·98	0·50	0·37, 0·63	0·11	0·07, 0·14	1·57	0·70, 2·44
Education level												
< Primary school (ref)												
Primary school	0·96	0·31, 1·61	0·24	0·09, 0·40	0·27	0·06, 0·48	0·32	0·13, 0·51	0·07	0·03, 0·11	1·23	–0·01, 2·48
Secondary school	0·99	0·27, 1·72	0·24	0·06, 0·41	0·27	0·05, 0·49	0·62	0·41, 0·83	0·11	0·06, 0·16	2·71	1·25, 4·17
University	1·57	0·76, 2·37	0·39	0·19, 0·60	0·46	0·20, 0·71	0·79	0·56, 1·03	0·13	0·07, 0·18	2·27	0·80, 3·74
Smoking												
Never (ref)												
Former	–0·23	–0·95, 0·49	–0·04	–0·22, 0·14	–0·02	–0·25, 0·20	–0·17	–0·36, 0·02	–0·03	–0·08, 0·03	–0·27	–1·56, 1·01
Current	–0·97	–1·78, –0·16	–0·22	–0·44, –0·003	–0·41	–0·75, –0·08	–0·41	–0·74, –0·08	–0·05	–0·11, 0·006	–1·06	–2·66, 0·54
Alcohol												
Never (ref)												
Former	0·29	–0·71, 1·29	0·09	–0·16, 0·34	0·06	–0·21, 0·34	–0·04	–0·34, 0·26	0·04	–0·06, 0·14	–0·56	–2·43, 1·31
Current	0·24	–0·43, 0·92	0·14	–0·03, 0·31	0·08	–0·13, 0·29	–0·19	–0·38, 0·01	–0·02	–0·08, 0·05	–1·31	–2·59, –0·03
Marital stat												
Single (ref)												
Married	0·29	0·96, 2·61	0·10	–0·06, 0·26	0·16	–0·03, 0·35	0·01	–0·17, 0·20	0·03	–0·008, 0·07	0·66	–0·41, 1·72
Separated	0·22	–1·29, 1·72	0·06	–0·29, 0·41	0·02	–0·48, 0·51	–0·47	–0·99, 0·06	–0·02	–0·12, 0·08	–1·67	–4·83, 1·48
Widow	–0·007	–1·13, 1·13	0·03	–0·25, 0·31	0·09	–0·25, 0·43	–0·40	–0·77, –0·04	0·003	–0·08, 0·08	0·04	–2·19, 2·27
Season												
Long dry (ref)												
Light rain	1·78	0·96, 2·61	0·39	0·19, 0·58	0·41	0·22, 0·61	–0·06	–0·27, 0·15	0·13	0·07, 0·18	–0·66	–1·91, 0·60
Short dry	0·53	0·004, 1·05	0·09	–0·05, 0·22	–0·01	–0·16, 0·14	–0·04	–0·06, 0·15	0·09	0·05, 0·13	–0·42	–1·44, 0·61
Heavy rain	–1·01	–1·60, –0·41	–0·24	–0·40, –0·08	–0·34	–0·56, –0·11	–0·36	–0·58, –0·14	–0·05	–0·09, –0·02	–1·91	–3·30, –0·53
Fruit (times/week)	0·04	–0·03, 0·11	0·01	–0·006, 0·03	0·01	–0·008, 0·03	–0·04	–0·06, –0·02	–0·002	–0·01, 0·003	–0·16	–0·29, –0·04
Vegetable (times/week)	0·12	0·05, 0·18	0·03	0·02, 0·05	0·04	0·02, 0·06	–0·06	–0·07, –0·04	–0·001	–0·005, 0·003	–0·22	–0·33, –0·11
PAEE (MJ/kg/d)	1·08	–9·7, 11·8	1·04	–1·63, 3·72	2·82	–0·04, 5·70	–6·87	–9·75, –4·0	–2·1	–4·31, 0·30	–39·0	–58·4, –19·6
Objective sedentary time (h/d)	0·05	–0·05, 0·15	0·0007	–0·002, 0·004	–0·02	–0·04, 0·01	0·04	0·01, 0·07	0·02	–0·003, 0·04	0·23	0·05, 0·41
Objective LPA (h/d)	–0·12	–0·27, 0·03	–0·003	–0·007, 0·001	–0·008	–0·05, 0·03	–0·03	–0·07, 0·01	–0·008	–0·02, 0·003	–0·07	–0·33, 0·19
Objective MVPA (h/d)	0·03	–0·13, 0·18	0·002	–0·002, 0·007	0·05	0·001, 0·09	–0·07	–0·11, –0·03	–0·03	–0·07, 0·006	–0·60	–89, –0·30
GPAQ PAEE (MJ/kg/d)	5·75	2·76, 8·74	1·68	0·96, 2·39	2·22	1·49, 0·2·95	–1·92	–2·63, –1·21	–0·66	–1·24, –0·08	–11·5	–16·9, –6·13
GPAQ work	0·08	0·04, 0·12	0·001	0·0006, 0·003	0·03	0·02, 0·04	–0·03	–0·04, –0·02	–0·01	–0·02, –0·003	–0·14	–0·22, –0·06
GPAQ leisure	–0·07	–0·21, 0·06	–0·0001	–0·0003, 0·0007	–0·007	–0·05, 0·04	0·03	–0·004, 0·06	0·02	–0·009, 0·05	–0·14	–0·47, 0·19
GPAQ travel	0·25	0·08, 0·42	0·0003	0·0001, 0·0005	0·10	0·05, 0·15	–0·10	–0·14, –0·06	–0·02	–0·05, 0·02	–0·70	–0·98, –0·42
BMI (kg/m2)	–0·009	–0·06, 0·04	–0·0005	–0·01, 0·01	–0·004	–0·02, 0·01	0·03	0·02, 0·05	0·003	–0·0002, 0·006	0·18	0·08, 0·29
Body fat %	0·006	–0·02, 0·04	0·004	–0·004, 0·01	0·002	–0·006, 0·01	0·02	0·01, 0·03	0·002	0·0004, 0·005	0·12	0·06, 0·18
Waist (cm)	–0·004	–0·02, 0·02	–0·0003	–0·005, 0·005	–0·002	–0·007, 0·004	0·01	0·005, 0·02	0·002	0·001, 0·0003	0·07	0·02, 0·11
HDL-cholesterol (mmol/l)	2·97	2·24, 3·71	0·75	0·57, 0·93	0·84	0·62, 1·07	0·26	0·19, 0·33	0·18	0·13, 0·24	5·63	4·29, 7·0
LDL-cholesterol	0·81	0·45, 1·17	0·17	0·08, 0·26	0·21	0·12, 0·30	0·15	0·1, 0·19	0·05	0·03, 0·07	2·8	2·16, 3·45
Total cholesterol (mmol/l)	0·92	0·62, 1·23	0·21	0·14, 0·29	0·25	0·17, 0·33	0·13	0·10, 0·17	0·05	0·04, 0·07	2·80	2·29, 3·31
TAG (mmol/l)	–0·22	–0·72, 0·28	–0·03	–0·15, 0·09	–0·04	–0·19, 0·10	–0·07	–0·12, –0·02	–0·04	–0·08, 0·003	0·58	–0·58, 1·74

PAEE, physical activity energy expenditure; GPAQ, Global Physical Activity Questionnaire; LPA, Light physical activity; MVPA, moderate-to-vigorous PA.

*β*-coefficient represents the difference in serum biomarkers in µmol/l per a unit difference in the predictor. Estimates are adjusted for age and sex (except for age adjusted for sex only and sex adjusted for age only).

**Figure 2.**
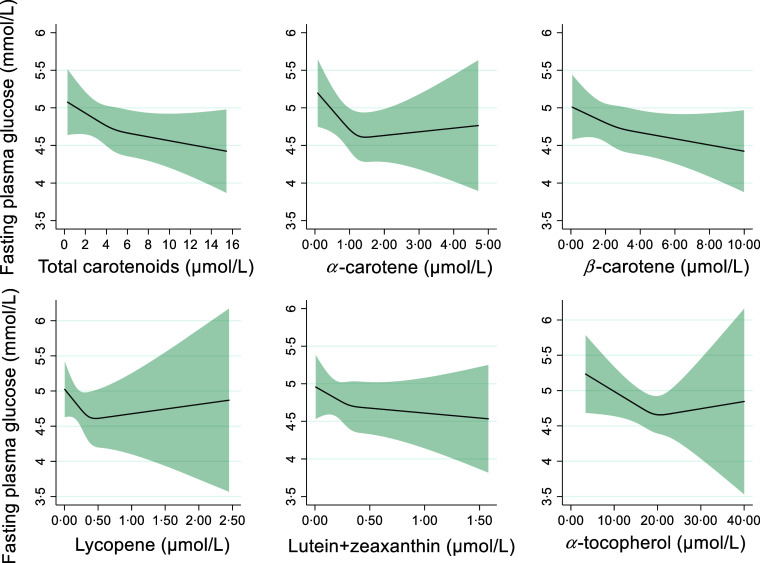
Associations of plasma carotenoids and *α*-tocopherol with fasting glucose, Cameroon Study, *n* 592. The shaded area represents the 95% confidence interval. Modelled using a restricted cubic spline function with three knots placed at 25th, 50th and 75th percentiles.

The associations of plasma carotenoids and *α*-tocopherol with fasting glucose are shown in Table 3. Plasma total carotenoids and *α*-carotene were significantly inversely associated with fasting glucose. Comparing the highest with the lowest tertile of the biomarkers, fasting glucose was lower by −0·28 (95 % CI −0·56, −0·001) mmol/l for plasma total carotenoids and −0·31 (–0·59, −0·03) mmol/l for plasma *α*-carotene. The inverse associations became stronger after adjusting for age, sex, education level, residential site, smoking status, alcohol intake, season, objectively measured physical activity, BMI and total cholesterol (–0·36 (–0·73, −0·002) mmol/l for total carotenoids and −0·41 (–0·79, −0·03) mmol/l for *α*-carotene). There was no evidence of an association of plasma concentrations of lycopene, lutein-zeaxanthin and *α*-tocopherol with fasting glucose. We did not find evidence of a non-linear association between any of the biomarkers and fasting glucose. The shapes of the associations are presented in Figure 2.

**Table 3.** Associations of plasma carotenoids and *α*-tocopherol with fasting glucose (mmol/l) (Cameroon study, *n* 592) Table 3 long description.

		Tertile 2		Tertile 3		*P*-value for linear trend
	Tertile 1	*β*-coefficient	95 % CI	*β*-coefficient	95 % CI	
Total carotenoids (range, µmol/l)	0·27, 3·39	3·4, 5·74		5·75, 18·94		
Model 1	1·0 (ref)	–0·27	–0·56, 0·03	–0·36	–0·65, –0·08	0·013
Model 2	1·0 (ref)	–0·26	–0·55, 0·02	–0·40	–0·71, –0·08	0·013
Model 3	1·0 (ref)	–0·28	–0·59, 0·03	–0·36	–0·73, –0·002	0·04
*α*-carotene (range, µmol/l)	0·08, 0·9	0·91, 1·47		1·48, 4·71		
Model 1	1·0 (ref)	–0·24	–0·54, 0·06	–0·38	–0·67, –0·10	0·008
Model 2	1·0 (ref)	–0·27	–0·56, 0·02	–0·46	–0·79, –0·14	0·005
Model 3	1·0 (ref)	–0·26	–0·59, 0·08	–0·41	–0·79, –0·03	0·03
*β*-carotene (range, µmol/l)	0·08, 1·8	1·81, 3·39		3·4, 11·03		
Model 1	1·0 (ref)	–0·11	–0·40, 0·19	–0·32	–0·61, –0·03	0·029
Model 2	1·0 (ref)	–0·12	–0·40, 0·16	–0·33	–0·66, –0·01	0·045
Model 3	1·0 (ref)	–0·13	–0·44, 0·18	–0·29	–0·67, 0·07	0·12
Lycopene (range, µmol/l)	0·005, 0·24	0·25, 0·47		0·48, 2·45		
Model 1	1·0 (ref)	–0·19	–0·48, 0·09	–0·18	–0·49, 0·12	0·251
Model 2	1·0 (ref)	–0·23	–0·53, 0·07	–0·31	–0·75, 0·12	0·157
Model 3	1·0 (ref)	–0·19	–0·52, 0·15	–0·25	–0·76, 0·25	0·32
Luteine + zeaxanthin (range, µmol/l)	0·007, 0·24	0·25, 0·38		0·39, 1·58		
Model 1	1·0 (ref)	–0·23	–0·52, 0·07	–0·22	–0·52, 0·08	0·265
Model 2	1·0 (ref)	–0·14	–0·42, 0·13	–0·19	–0·47, 0·08	0·165
Model 3	1·0 (ref)	–0·11	–0·40, 0·18	–0·16	–0·46, 0·14	0·28
*α*-tocopherol (range, µmol/l)	0·68, 17·24	17·25, 21·67		21·68, 48·01		
Model 1	1·0 (ref)	–0·23	–0·52, 0·06	–0·26	–0·60, 0·08	0·099
Model 2	1·0 (ref)	–0·27	–0·52, –0·03	–0·33	–0·67, 0·01	0·053
Model 3	1·0 (ref)	–0·22	–0·47, 0·02	–0·29	–0·67, 0·09	0·14

*P*-values for trend are from a linear regression model for continuous variables including tertiles of the biomarkers as a continuous exposure.

Model 1: Adjusted for age and sex.

Model 2: Model 1 + education level, residential site (4 sites), smoking, alcohol intake, season of data collection (4 seasons) + objectively measured physical activity (continuous) + BMI (continuous).

Model 3: Model 2 + total cholesterol.

There was no evidence of an effect modification by sex, rural–urban area of residence, BMI categories and smoking status on the associations of plasma carotenoids and *α*-tocopherol with fasting glucose. Results were unchanged in sensitivity analyses using multiple imputation to account for the effect of missing covariates.

## Discussion

In this population-based cross-sectional study in rural and urban Cameroon, higher concentrations of plasma total carotenoids and *α*-carotene were associated with lower fasting glucose, independently of sociodemographic characteristics and health-related behaviours. This study provides for the first time, to our knowledge, the relationship between intake of fruit and vegetables, quantified objectively using biomarkers, and fasting glucose in a population-based study in adults in Africa.

The distribution of plasma carotenoids in this study differed from those in previous studies. While plasma *α*- and *β*-carotene constituted about 85 % of total carotenoids in our study, they contribute only about 35 % in the NDNS in the UK. In absolute concentrations, the means of plasma *α*- and *β*-carotene in this study were over 4-fold higher than those reported in the NDNS and National Health and Nutrition Examination Survey (NHANES) in the USA^(25,26)^ but were lower than *α*- and *β*-carotene concentrations reported in a study in pregnant women in Nigeria^(27)^. On the other hand, the means of plasma lycopene, lutein and zeaxanthin concentrations were similar to those reported in the NHANES but lower than in the NDNS, where lycopene, lutein and zeaxanthin were the leading contributors to plasma total carotenoids concentrations^(25,26)^.

In this study, we observed positive correlations between plasma total carotenoids and self-reported intake of fruits (*r* = 0·13) and vegetables (*r* = 0·29). Similarly, *β*-carotene was positively correlated with fruit (*r* = 0·17) and vegetable (*r* = 0·35) intake, while *α*-carotene showed positive correlations with fruit (*r* = 0·14) and vegetable (*r* = 0·32) intake. These correlation coefficients are comparable to those reported in other populations. For example, a study among non-smokers in the USA reported positive correlations between combined fruit and vegetable intake and total carotenoids (*r* = 0·34) as well as *β*-carotene (*r* = 0·35)^(10)^. Likewise, findings from the European Prospective Investigation into Cancer (EPIC) study demonstrated positive correlations between total carotenoids and fruit (*r* = 0·61) and vegetable (*r* = 0·37) intake assessed using an FFQ^(12)^. In our study, correlations between biomarkers and self-reported intake were consistently stronger for vegetable than for fruit intake, which may reflect the higher frequency of vegetable intake relative to fruit intake in this population.

The inverse association of plasma lycopene with self-reported intake of fruit and vegetables is not well understood. In Western settings, tomato and tomato-based food products are the main sources of lycopene^(28)^. However, biomarker concentrations are affected by food storage and cooking methods. A study amongst African Americans showed that cooked tomatoes and watermelon contributed to a substantial proportion of dietary lycopene intake, and raw tomatoes contributed little^(28)^. In addition to the potential misreporting of tomatoes intake, the inverse association between plasma lycopene and self-reported intake of fruit and vegetables may suggest the contribution of other food sources to plasma lycopene concentrations, such as palm oils in this population. We found an inverse association of intakes of fruit and vegetables with *α*-tocopherol, after adjusting for age and sex. Although considerable amounts of *α*-tocopherol are found in certain fruits and vegetables, their main dietary sources are vegetable oils^(29)^.

Consistent with smoking being a major determinant of carotenoids concentrations, we found that current smokers had lower plasma total carotenoids concentrations than participants who had never smoked^(30–31)^. Cigarette smoking contains free radicals, which induce oxidative stress, leading to increased utilisation of antioxidants such as carotenoids^(32)^.

Our findings of an inverse association of plasma total carotenoids and *α*-carotene with fasting glucose are consistent with previous observational studies^(33–36)^. In a meta-analysis of thirteen prospective observational studies, higher circulating total carotenoids were associated with lower risk of type 2 diabetes^(35)^. In contrast, other cross-sectional and prospective cohort studies reported no evidence of an association between circulating carotenoids and glycaemic markers, or type 2 diabetes risk^(30,31,37)^.

In this study, plasma total carotenoids and *α*- and *β*-carotene were positively correlated with self-reported fruit and vegetable intake, and the magnitude was comparable to those reported in previous studies^(9,12)^. Therefore, our finding of an inverse association between plasma total carotenoids and fasting glucose may suggest an inverse association between intake of fruits and vegetables and fasting glucose. A meta-analysis of twenty-three prospective observational studies showed that higher intake of fruit and vegetables was associated with lower risk of type 2 diabetes^(7)^. We showed an inverse association between plasma *α*-carotene and fasting glucose, which may suggest an association of carrots and root vegetables with fasting glucose, given that plasma *α*-carotene has been shown to highly correlate with intake of carrots and root vegetables^(12)^.

The potential mechanisms of action for intake of fruit and vegetables on diabetes could be that fruit and vegetables help in weight loss, since they are rich in fibres, help to increase satiety and are low-energy-density foods^(38)^. This is consistent with our results showing an inverse association between self-reported fruit and vegetable intake and BMI. Besides being rich sources of carotenoids and tocopherol, fruit and vegetables are also rich in other vitamins, like vitamin C, folate, minerals like Mg and phytochemicals like polyphenols, all of which have been shown to lower systemic inflammation and oxidative stress, processes involved in the development and progression of type 2 diabetes^(39,40)^. Dietary fibres and polyphenols found in fruit and vegetables may modulate the gut microbiome^(41)^. An altered gut microbiome has been associated with a higher risk of type 2 diabetes^(42)^.

We did not find evidence of associations of plasma lycopene, lutein-zeaxanthin and *α*-tocopherol with fasting glucose, which is consistent with previous studies^(16,30,33,34,43–45)^. In prospective cohort studies in the USA and Finland, there was no evidence of an association between dietary lycopene or intake of tomato-based food and type 2 diabetes risk^(4,44)^. We observed a significant inverse association between plasma *α*-carotene and fasting glucose and a borderline inverse association for *β*-carotene, which are provitamin A carotenoids. Previous studies have also reported an inverse association between provitamin A carotenoids and diabetes but no association for non-provitamin A carotenoids^(14,34)^. This could be because provitamin A carotenoids are converted in the body to retinol and may improve insulin sensitivity by binding to retinol-binding protein 4 (RBP4), the only specific binding protein for retinol. High serum RBP4 has been associated with higher insulin resistance in people with obesity and type 2 diabetes^(46)^.

Evidence of an inverse association between dietary or plasma carotenoids comes mainly from observational studies, whereas intervention studies mainly show no beneficial effect. In the Alpha-Tocopherol, Beta-Carotene Cancer Prevention Study, supplementation with *β*-carotene at 20 mg/d or *α*-tocopherol at 50 mg/d in male smokers did not reduce the risk of type 2 diabetes after 6·1 years^(47)^. Compared with placebo, supplementation with *β*-carotene (50 mg every other day), *α*-tocopherol (600 IU every other day) or vitamin C (500 mg/d) in the Women’s Antioxidant Cardiovascular Study had no effect on type 2 diabetes risk after 9·2 years^(48)^. Similarly, supplementation with *β*-carotene at 50 mg every other day did not reduce the risk of diabetes in the Physician’s Health Study after 12 years^(49)^. This could be because the observed inverse association between carotenoid levels and type 2 diabetes risk in observational studies may reflect the benefits of fruits and vegetables rather than a direct effect of carotenoids themselves. Moreover, fruits and vegetables are rich in different micronutrients and non-nutritive compounds like flavonoids, and it is possible that the benefits observed result from a synergistic effect. This could also provide an explanation for the null association observed in some prospective observational studies of dietary carotenoids intake and risk of type 2 diabetes^(4,50)^. Moreover, studies using the Mendelian randomisation approach suggest no causal association between circulating *β*-carotene or vitamin C and type 2 diabetes^(51,52)^. Future studies are needed to identify the contributions of different foods to carotenoids concentrations in this population.

### Strengths and limitations

The main strength of this study relies on the use of objectively measured biomarkers to assess intake of fruit and vegetables, to reduce the measurement error and recall bias associated with the self-reports. These analyses were conducted in a population-based study, with participants from rural and urban settings of Cameroon, and we adjusted for potential confounders, including objectively measured physical activity.

The major limitation of this study is the cross-sectional study design, which limits our ability to infer a causal effect, due to the possibility of reverse causation. We used plasma carotenoids and *α*-tocopherol as objective indicators of intake of fruit and vegetables, but their bioavailability may be influenced by intrinsic and extrinsic factors, such as genetic variations, food composition, processing and storage. The concentration of the provitamin A carotenoids may also be influenced by the rate of conversion to vitamin A which is dependent on vitamin A status. Moreover, carotenoids and tocopherol are antioxidants, and their concentrations may be affected by underlying health conditions^(53,54)^. While we controlled for potential confounders, we cannot rule out the possibility of residual confounding in the associations observed. This study was conducted in young adults, and our findings may not be generalisable to other age ranges, or even outside this geographical location. Another limitation is that we were unable to measure vitamin C in the same samples. The samples were stored without a stabilising agent like metaphosphoric acid, and therefore we were unable to detect meaningful concentrations of vitamin C in these samples.

### Conclusion

In this population-based study, higher total carotenoids, specifically *α*-carotene, as objective indicators of fruit and vegetable intake, were associated with lower fasting glucose, independently of sociodemographic characteristics and health-related behaviours. These findings add to the evidence in favour of the public health recommendation to increase fruits and vegetable intake, to prevent diabetes in this setting and more widely in African populations in whom the intake of fruit and vegetables is low.

## Supporting information

Mba et al. supplementary material Mba et al. supplementary material

## Supplementary material

For supplementary material/s referred to in this article, please visit https://doi.org/10.1017/S0007114526106503

## Table 1 Long description

A table with 8 rows and 8 columns showing pairwise correlations of individual biomarkers and fruit and vegetable intake. The columns are labeled as follows: α-carotene, β-carotene, Lycopene, Lutein + zeaxanthin, α-tocopherol, Fruit intake, and Vegetable intake. The rows are labeled as follows: Total carotenoids, α-carotene, β-carotene, Lycopene, Lutein-zeaxanthin, α-tocopherol, and Fruit. Each cell contains a correlation coefficient value. Row 1: Total carotenoids, α-carotene 0.97, β-carotene 0.98, Lycopene 0.46, Lutein + zeaxanthin 0.62, α-tocopherol 0.47, Fruit intake 0.13, Vegetable intake 0.29. Row 2: α-carotene, β-carotene 0.95, Lycopene 0.38, Lutein + zeaxanthin 0.56, α-tocopherol 0.41, Fruit intake 0.14, Vegetable intake 0.32. Row 3: β-carotene, Lycopene 0.32, Lutein + zeaxanthin 0.53, α-tocopherol 0.39, Fruit intake 0.17, Vegetable intake 0.35. Row 4: Lycopene, Lutein + zeaxanthin 0.42, α-tocopherol 0.57, Fruit intake -0.12, Vegetable intake -0.29. Row 5: Lutein-zeaxanthin, α-tocopherol 0.47, Fruit intake 0.06, Vegetable intake 0.13. Row 6: α-tocopherol, Fruit intake -0.04, Vegetable intake -0.03. Row 7: Fruit, Vegetable intake 0.32.

Navigate back to Table 1.

## Table 2 Long description

A table with 592 rows and 21 columns showing factors affecting plasma carotenoids and tocopherol in a Cameroon study. The table includes biomarkers such as total carotenoids, alpha-carotene, beta-carotene, lycopene, lutein plus zeaxanthin, and alpha-tocopherol. Each biomarker has a beta-coefficient and 95 percent confidence interval. The table is divided into various factors like age, sex, urban vs. rural, education level, smoking status, alcohol consumption, marital status, season, fruit and vegetable intake, physical activity, body measurements, and cholesterol levels. Each row represents a different factor and its association with the biomarkers. Notable trends include positive associations with age, urban living, education level, light rain season, vegetable intake, physical activity, and cholesterol concentrations. Negative associations include male sex, heavy rain season, current smoking, and BMI.

Navigate back to Table 2.

## Table 3 Long description

A table comparing the associations of plasma carotenoids and alpha-tocopherol with fasting glucose levels. The table has 6 rows and 11 columns. Column headers are Total carotenoids (range, µmol/l), alpha-carotene (range, µmol/l), beta-carotene (range, µmol/l), Lycopene (range, µmol/l), Luteine + zeaxanthin (range, µmol/l), and alpha-tocopherol (range, µmol/l). Row labels are Tertile 1, Tertile 2, Tertile 3, Model 1, Model 2, and Model 3. Row 1: Total carotenoids (range, µmol/l), 0-27, 3-39, 5-75, 18-94. Row 2: alpha-carotene (range, µmol/l), 0-08, 0-9, 1-48, 4-71. Row 3: beta-carotene (range, µmol/l), 0-08, 1-8, 3-4, 11-03. Row 4: Lycopene (range, µmol/l), 0-005, 0-24, 0-25, 0-47, 0-48, 2-45. Row 5: Luteine + zeaxanthin (range, µmol/l), 0-007, 0-24, 0-25, 0-38, 0-39, 1-58. Row 6: alpha-tocopherol (range, µmol/l), 0-68, 17-24, 17-25, 21-67, 21-68, 48-01. Each row contains beta-coefficient values and 95 percent confidence intervals for Models 1, 2, and 3, along with P-values for linear trend.

Navigate back to Table 3.
