# Supplementary material for: The associations of plasma carotenoids and α-tocopherol concentrations with fasting glucose in Cameroon
Source: Br J Nutr. 2026 Feb 12;135(11):1153–63. doi: 10.1017/S0007114526106503 (PMC13423521; doi:10.1017/S0007114526106503)
Supplement: Mba et al. supplementary material [file S0007114526106503sup001.docx]

Supplementary material:

Eligible participants (n = 3854)

Rural: n = 2238, mean age = 35.3 ± 7.7 y

Urban: n = 1616, mean age = 35.4 ± 8.3 y

651 volunteers

Rural: n = 303, age = 38.5 ± 8.3 y

Urban: n = 348, age = 37.9 ± 9.1 y

No blood samples available n = 59

592 (carotenoids and tocopherol measurement)

Rural, n = 272, age= 38.8 ± 8.2 y

Urban, n = 320, age= 37.5 ± 8.9 y

Supplementary Figure 1: Work flow chart

Supplementary Table 1: Socio-demographic and metabolic characteristics of the study population stratified by sex and urban/rural residence (Cameroon study: n=592)

|  | Women (n=372) | | | Men (n=220) | | |
| --- | --- | --- | --- | --- | --- | --- |
|  | Rural (n=173) | Urban (n=199) | p-value | Rural (n=104) | Urban (n=116) | p-value |
| Age (years) | 39.8±8.1 | 38.6±8.8 | 0.168 | 37.3±8.3 | 36.3±8.9 | 0.368 |
| Education (years) | 8.0±4.1 | 11.8±5.2 | <0.001 | 9.0±4.5 | 13.2±5.2 | <0.001 |
| Education, n (%)  < Primary  Completed primary  Secondary and high school  University | 53(30.6)  93(53.8)  24(13.9)  3(1.7) | 24(12.0)  75(37.7)  66(33.2)  34(17.1) | < 0.001 | 22(22.2)  57(57.6)  16(16.2)  4(4.0) | 4(3.5)  34(29.8)  41(36.0)  35(30.7) | < 0.001 |
| Alcohol intake, n (%)  Never  Past  Current | 26(15.0)  17(9.8)  173(75.2) | 24(12.1)  26(13.1)  149(74.8) | 0.485 | 10(10.0)  03(3.0)  87(87.0) | 06(5.3)  10(8.7)  98(86.0) | 0.104 |
| Smoking status, n (%):  Never  Past smoker  Current smoker | 161(93.1)  11(6.4)  1(0.6) | 182(91.5)  16(8.0)  1(0.5) | 0.820 | 57(57.0)  21(21.0)  22(22.0) | 54(47.4)  31(27.2)  29(25.4) | 0.357 |
| Fruit (times/week) | 3(2-6) | 2(1-5) | 0.108 | 2(1-6) | 2(1-3) | 0.028 |
| Vegetable (times/week) | 6(3-9) | 4(2-6) | <0.0001 | 4(2-6) | 3(2-6) | 0.153 |
| Fruit and vegetable, n (%)  < 3 times/ week  3-6 times/ week  ≥ 7 times/ week | 7(4.3)  41(25.2)  115(70.6) | 15(8.2)  74(40.2)  95(51.6) | 0.001 | 12(12.6)  29(30.5)  54(56.8) | 16(14.8)  53(49.1)  39(36.1) | 0.01 |
| PAEE (KJ/Kg/day) | 54.3±20.9 | 38.3±16.6 | <0.0001 | 65.9±26.2 | 52.1±22.6 | 0.0001 |
| BMI (kg/m^2^) | 24.9±4.9 | 29.2±5.5 | <0.0001 | 23.09±3.23 | 25.3±4.0 | <0.0001 |
| Waist circumference(cm) | 85.2±11.5 | 94.3±12.9 | <0.0001 | 82.9±7.9 | 89.2±11.3 | <0.0001 |
| Systolic blood pressure (mmHg) | 117.1±18.8 | 123.8±21.9 | 0.002 | 120.5±15.1 | 130.4±22.9 | 0.0003 |
| Diastolic Blood Pressure (mmHg) | 74.2±12.0 | 79.6±13.7 | <0.0001 | 72.3±11.9 | 78.0±14.6 | 0.002 |
| Fasting blood glucose (mmol/L) | 4.81±1.39 | 4.86±1.08 | 0.682 | 4.72±1.49 | 4.63±1.55 | 0.676 |
| Total cholesterol (mmol/L) | 3.84±0.95 | 4.02±0.98 | 0.08 | 3.60±0.90 | 3.80±0.90 | 0.318 |
| LDL cholesterol (mmol/L) | 2.26±0.83 | 2.36±0.85 | 0.23 | 2.07±0.79 | 2.15±0.85 | 0.363 |
| HDL cholesterol (mmol/L) | 1.18±0.33 | 1.27±0.32 | 0.017 | 1.19±0.35 | 1.23±0.31 | 0.362 |
| Triglycerides (mmol/L) | 0.78(0.63-1.01) | 0.71(0.57-0.90) | 0.026 | 0.70(0.58-0.94) | 0.73(0.56-0.99) | 0.876 |

Results are presented as arithmetic mean [or median (25th-75th percentile) for non-normally distributed variables] or n (%). p-values are from a t-test for normally distributed continuous variables (or Mann Whitney test for non-normally distributed variables) and from a chi squared test for categorical variables.

PAEE, physical activity energy expenditure; BMI, body mass index; LDL cholesterol, low-density lipoproteins cholesterol; HDL cholesterol, high-density lipoproteins cholesterol;

Supplementary Figure 2: Contribution of plasma individual carotenoids to total carotenoids in the Cameroon study (Compared with the National Diet and Nutrition Survey year 1-4 combined) (26)

Supplementary Table 2: Correlates of self-reported fruit and vegetables intake (Cameroon study, n=592)

|  | self-reported fruit intake (times/week)  β (95% confidence interval) | self-reported vegetable intake (times/week)  β (95% confidence interval) |
| --- | --- | --- |
| Age (10 y) | 0.04(-0.3 to 0.4) | 0.8(0.5 to 1.2) |
| men | -0.85(-1.42 to -0.28) | -1.11(-1.75 to -0.47) |
| Urban (vs rural) | -1.01(-1.58 to -0.44) | -1.54(-2.18 to -0.89) |
| Education level  <primary school (ref)  primary school  Secondary school  University | 0.11(-0.75 to 0.98)  -0.09(-0.97 to 0.79)  0.22(-0.84 to 1.29) | -0.69(-1.69 to 0.31)  -1.19(-2.26 to -0.12)  -0.95(-2.07 to 0.17) |
| Smoking  Never (ref)  Former  Current | -0.11(-0.91 to 0.70)  0.38(-0.74 to 1.50) | -0.71(-1.53 to 0.11)  -0.52(-1.59 to 0.55) |
| Alcohol  Never (ref)  Former  Current | 0.93(-0.29 to 2.15)  0.95(0.07 to 1.83) | 0.18(-1.13 to 1.49)  0.55(-0.49 to 1.59) |
| Marital stat  Single (ref)  Married  Separated  Widow | 0.40(-0.37 to 1.18)  1.11(-0.40 to 2.62)  -0.23(-1.54 to 1.07) | 0.68(-0.10 to 1.47)  2.25(0.11 to 4.38)  2.49(0.92 to 4.07) |
| Family size  <3 (ref)  3-5  >5 | 0.45(-0.21 to 1.11)  0.69(-0.22 to 1.61) | -0.28(-1.05 to 0.49)  -0.79(-1.76 to 0.18) |
| Season  Long dry (ref)  Light rain  Short dry  Heavy rain | 1.65(0.57 to 2.72)  0.46(-0.15 to 1.08)  0.30(-0.58 to 1.17) | 1.74(0.64 to 2.84)  0.31(-0.41 to 1.03)  0.51(-0.70 to 1.72) |
| PAEE (MJ/kg/d) | 22.0(7.80 to 36.2) | 38.08(22.62 to 53.54) |
| Objective sedentary (h/day) | -0.13(-0.26 to -0.009) | -0.22(-0.36 to -0.09) |
| Objective LPA (h/day) | 0.10(-0.07 to 0.28) | 0.12(-0.06 to 0.31) |
| Objective MVPA (h/day) | 0.25(0.02 to 0.48) | 0.50(0.26 to 0.74) |
| GPAQ PAEE (MJ/kg/d) | 4.63(1.09 to 8.16) | 15.53(11.21 to 19.86) |
| GPAQ work | 0.06(0.01 to 0.11) | 0.19(0.13 to 0.26) |
| GPAQ leisure | -0.18(-0.30 to -0.05) | -0.08(-0.30 to 0.13) |
| GPAQ travel | 0.21(0.009 to 0.41) | 0.91(0.68 to 1.14) |
| BMI (kg/m^2^) | -0.06(-0.11 to -0.006) | -0.14(-0.20 to -0.08) |
| Body fat % | -0.03(-0.07 to 0.005) | -0.08(-0.12 to -0.04) |
| waist | -0.02(-0.04 to 0.007) | -0.04(-0.06 to -0.01) |

β-coefficient represents the difference in in the frequency of intakes of fruits and vegetables per a unit difference in the predictor. Estimates are adjusted for age and sex (except for age adjusted for sex only and sex adjusted for age only)

PAEE, physical activity energy expenditure; LPA, Light physical activity; MVPA, moderate to vigorous PA; BMI, body mass index
